# *Kosakonia cowanii* Ch1 Isolated from Mexican Chili Powder Reveals Growth Inhibition of Phytopathogenic Fungi

**DOI:** 10.3390/microorganisms11071758

**Published:** 2023-07-05

**Authors:** Jacqueline González Espinosa, Yoali Fernanda Hernández Gómez, Yomaiko Javier Martínez, Francisco Javier Flores Gallardo, Juan Ramiro Pacheco Aguilar, Miguel Ángel Ramos López, Jackeline Lizzeta Arvizu Gómez, Carlos Saldaña Gutierrez, José Alberto Rodríguez Morales, María Carlota García Gutiérrez, Aldo Amaro Reyes, Erika Álvarez Hidalgo, Jorge Nuñez Ramírez, José Luis Hernández Flores, Juan Campos Guillén

**Affiliations:** 1 Facultad de Ciencias Naturales, Universidad Autónoma de Querétaro, Av. De las Ciencias S/N, Querétaro 76220, Mexico; jgonzalez111@alumnos.uaq.mx (J.G.E.); yhernandez01@alumnos.uaq.mx (Y.F.H.G.); carlos.saldana@uaq.mx (C.S.G.); 2 Facultad de Química, Universidad Autónoma de Querétaro, Cerro de las Campanas S/N, Querétaro 76010, Mexico; yomaiko_javier@outlook.com (Y.J.M.); javi_96_5@hotmail.com (F.J.F.G.); ramiro.pacheco@uaq.mx (J.R.P.A.); agromyke@gmail.com (M.Á.R.L.); maria.carlota.garcia@uaq.edu.mx (M.C.G.G.); aldo.amaro@uaq.edu.mx (A.A.R.); erialvarez@yahoo.com (E.Á.H.); jorge.nunez@uaq.mx (J.N.R.); 3 Secretaría de Investigación y Posgrado, Centro Nayarita de Innovación y Transferencia de Tecnología (CENITT), Universidad Autónoma de Nayarit, Tepic 63173, Mexico; lizzeta28@gmail.com; 4 Facultad de Ingeniería, Universidad Autónoma de Querétaro, Cerro de las Campanas S/N, Querétaro 76010, Mexico; josealberto970@hotmail.com; 5 Centro de Investigación y de Estudios Avanzados del IPN, Irapuato 36824, Mexico

**Keywords:** *Kosaconia cowanii* Ch1, volatile organic compounds, chili powder, fungal pathogens, biocontrol, genome sequencing

## Abstract

*Kosakonia cowanii* strain Ch1 was isolated from Mexican chili powder, and the genome was sequenced. The genome was 4,765,544 bp in length, with an average G + C content of 56.22%, and a plasmid (pCh1) of 128,063 bp with an average G + C content of 52.50%. A phylogenetic analysis revealed a close relation with pathogenic strains; nevertheless, some virulence-related genes were absent, and this genetic characteristic may explain the fact that *K. cowanii* Ch1 behaved as a non-pathogenic strain when infection assays were performed on the leaves and fruits of *Capsicum annuum* L. Surprisingly, we observed that this bacterial strain had the ability to spread throughout serrano pepper seeds. Furthermore, *K. cowanii* Ch1 was evaluated for the production of volatile organic compounds (VOCs) against fungal pathogens, and the results showed that *Alternaria alternata* and *Sclerotium rolfsii* were inhibited in a radial mycelial growth assay by a mean rate of 70% and 64%, while *Fusarium oxysporum* was inhibited by only approximately 10%. Based on the headspace solid-phase microextraction combined with the gas chromatography mass spectrometry (HS-SPME-GC-MS), 67 potential VOCs were identified during the fermentation of *K. cowanii* Ch1 in TSA medium. From these VOCs, nine main compounds were identified based on relative peak area: dodecanoic acid; 3-hydroxy ethanol; 1-butanol-3-methyl; acetaldehyde; butanoic acid, butyl ester; cyclodecane; 2-butanone, 3-hydroxy; disulfide, dimethyl and pyrazine-2,5-dimethyl. Our findings show the potential of *K. cowanii* Ch1 for the biocontrol of fungal pathogens through VOCs production and reveal additional abilities and metabolic features as beneficial bacterial specie.

## 1. Introduction

*Kosakonia cowanii* belongs to the *Enterobacteriaceae* family and was reclassified from the *Enterobacter* genus based on multilocus sequence analysis (MLSA) one decade ago [1]. It is a Gram-negative, facultative anaerobic, rod-shaped, and motile bacterium with a huge metabolic ability to colonize diverse environments. From this perspective and because of the presence of virulence factors associated with the *Kosakonia* genus, several reports indicate that some strains have been involved in human infection as rare events, and therefore, are assumed to be able to act as facultative human pathogens [2]. In this way, *K. radicincitans* has been isolated from a human bloodstream infection [3], as well as a sporadic case of a *K. cowanii* infection associated with an acute cholecystitis [4].

In recent years, *K. cowanii* has also emerged as an important phytopathogen causing diverse plant diseases [2]. For instance, some reports show the association of *K. cowanii* with bacterial wilt in tomatoes and patchouli plants [5], where typical symptoms of the disease cause the collapse of the plant [6]. Additionally, *K. cowanii* has been reported in *Eucalyptus* trees displaying symptoms of bacterial blight [7]. Another report showed that it possesses the ability to cause necrotic spots on leaves of *Mabea fistulifera* Mart. (*Euphorbiaceae*) [8]. Additionally, it has been detected in plants that present symptoms of bacterial stalk and leaf necrosis, such as onion (*Allium cepa* L.) [9], and as an emergent bacterial pathogen affecting soybean (*Glycine max* Willd) [6].

However, contrary to these adverse traits reported in the *Kosakonia* genus, in recent decades the increased characterization of microbiomes associated with plants and the isolation of beneficial microorganisms as inoculants to ensure crop yield and nutritional quality have allowed for the isolation of several strains of the *Kosakonia* genus as a plant growth-promoting rhizobacteria (PGPR), conferring upon plants the ability to tolerate abiotic stress [10,11] or as a biocontrol of phytopathogenic fungi [12]. Interestingly, one report showed that *K. cowanii* colonizing the gut of *Anopheles gambiae* displayed direct anti-*Plasmodium* properties [13]; therefore, these findings revealed that many metabolic features of the *Kosakonia* genus still remain unexplored [10]. Previous efforts to determine the bacterial diversity in Mexican chili powder [14] led us to detect the presence of *K. cowanii* by 16S rDNA Illumina sequencing, and several bacterial isolates were obtained. In this work, we present the results of the characterization of a new *K. cowanii* Ch1 strain through genome sequencing and phytopathogenicity testing in *Capsicum annuum* L. More importantly, we identify for the first time its ability to inhibit phytopathogenic fungi growth by volatile organic compounds (VOCs). *K. cowanii* Ch1 exhibits important biocontrol activity and could be a promising element for the biocontrol of economically important phytopathogenic microorganisms.

## 2. Materials and Methods

### 2.1. Kosakonia Strain Isolation

*K. cowanii* Ch1 was isolated from Mexican chili powder and identified by the Laboratory of Molecular Biology of Microorganisms at the Faculty of Chemistry of Queretaro University, Mexico [14]. *K. cowanii* Ch1 possesses a TEM β-lactamase (class A); therefore, the strain was grown on a tryptic soy agar (TSA) medium (Difco Laboratories, Detroit, MI, USA) supplemented with 100 µg/mL of ampicillin and incubated at 37 °C for 24 h. Additionally, antibiotic susceptibilities were determined using the criteria of CLSI standards [15]. *Escherichia coli* XL1 blue was used as a control. Antibiotic discs (Oxoid) containing amikacin (30 μg), ampicillin (10 μg), amoxicillin/clavulanic acid (20/10 µg), carbenicillin (100 µg), cephalothin (30 µg), cefotaxime (30 μg), chloramphenicol (30 μg), ciprofloxacin (5 μg), clindamycin (30 µg), dicloxacillin (1 µg), erythromycin (15 μg), gentamicin (10 μg), imipenem (10 µg), meropenem (10 µg), netilmicin (30 µg), nitrofurantoin (300 µg), norfloxacin (10 µg), penicillin (10 U), tetracycline (30 μg), trimethoprim-sulfamethoxazole (25 μg), and vancomycin (30 µg) were used.

### 2.2. Pathogenicity Test

The pathogenicity test of *K. cowanii* Ch1 was conducted by inoculating the bacterium on *Capsicum annuum* L. plantlets and fruits. *Capsicum annuum* L. var. serrano seeds were surface sterilized by immersion in 10% (*v*/*v*) sodium hypochlorite for 5 min and rinsed 3 times with sterile distilled water and germinated in seedling trays containing sterile mixed soil and placed under greenhouse condition. Then, seedlings were transplanted into pots containing sterile soil and grown under greenhouse conditions; 45 days post germination, seedlings with 12–14 true leaves were used. Fruits of the serrano pepper (*Capsicum annuum* L.) were selected based on size uniformity and the absence of visual damage or infections. Additionally, fruits were superficially disinfected by immersion in 10% (*v*/*v*) sodium hypochlorite for 10 min, then with 50% (*v*/*v*) ethanol for 10 min, and finally rinsed with sterile water and air-dried at room temperature (25 °C). For the bacterial inoculation test, *K. cowanii* Ch1 was grown in tryptic soy broth and incubated at 37 °C for 24 h with continuous shaking (100 rpm), and then the bacterial suspension was diluted to approximately 1 × 10^8^ CFU/mL. From this bacterial suspension, 10 plants were inoculated with 10 µL, using 5–6 leaves of each plant and 10 serrano pepper fruits; all were punctured with a sterile needle and inoculated at that point. Inoculated seedlings were grown in greenhouse conditions, while inoculated fruits were placed inside an airtight container at room temperature (25 °C) for 7 days. Negative controls were inoculated with sterile distilled water.

### 2.3. 16s rRNA Gene Sequence Analysis

Serrano pepper fruits inoculated with *K. cowanii* Ch1 and controls were analyzed for 7 days post inoculation, and seeds were obtained and placed on a TSA medium (Difco Laboratories; Detroit, MI, USA) supplemented with 100 µg/mL ampicillin and incubated at 37 °C for 24 h. Bacterial colonies that were grown close to the seeds were analyzed by PCR using primers 27F and 1492R for 16s rRNA [16]. The amplicons obtained were sequenced at Macrogen Inc. (Seoul, Republic of Korea) and analyzed using MEGA X with the neighbor-joining method. The evolutionary distances were computed using the Jukes-Cantor method and compared with the sequence of *K. cowanii* Ch1 [17,18,19,20,21].

### 2.4. Genome Sequencing and Assembly

Genomic DNA of the *K. cowanii* Ch1 strain was extracted using a ZymoBIOMICS^TM^ DNA Miniprep Kit (Zymo Research, Irvine, CA, USA) according to the manufacturer’s instructions. The genomic DNA was processed and analyzed with the Shotgun Metagenomic Sequencing Service (Zymo Research, Irvine, CA, USA). Sequencing libraries were prepared with an Illumina^®^ DNA Library Prep Kit (Illumina, San Diego, CA, USA), and the final library was sequenced on the platform NovaSeq^®^ (Illumina, San Diego, CA, USA). Bioinformatic analyses were made using the pipelines at the Bacterial and Viral Bioinformatics Resource Center (BV-BRC); the Fastq Utilities Service [22], the comprehensive genome analysis service at PATRIC [22], the RAST tool kit (RASTtk) for genome annotation [23], and CIRCOS for the circular genome map [24]. The Codon Tree pipeline of BV-BRC was used to generate bacterial phylogenetic trees. The closest reference genomes were identified using Mash/MinHash [25], and PATRIC global protein families (PGFams) [22] were selected and aligned with MUSCLE v5 [20], then concatenated into a data matrix, and RaxML v8.2.11 (Randomized Axelerated Maximum Likelihood) [21] was used to analyze the matrix with 100 rounds of fast bootstrapping options [22] to determine the phylogenetic analysis. Plasmid assembly was performed using the PlasmidSPAdes version v3.13.0 with default settings [26]. DNA sequences were deposited in NCBI as BioProject ID PRJNA965885. The genome accession number was JAUDFU000000000.

### 2.5. Inhibitory Effects of VOCs Produced by K. cowanii Ch1 on Pathogenic Fungi In Vitro

The following fungal pathogens were selected to test the inhibitory effects of *K. cowanii* Ch1 volatile organic compounds (VOCs): *Alternaria alternata*, which causes black spot in diverse fruits and vegetables around the world; *Fusarium oxysporum*, with diverse members of this species causing complex infections on economically relevant crops worldwide with typical symptoms such as chlorosis, necrosis of vascular systems, and potential death of colonized plants; and *Sclerotium rolfsii*, which is a very common soil-borne fungus infecting a wide range of vegetable, ornamental, and field crops. These fungal strains were provided by the Laboratory of Plants and Agriculture Biotechnology at Queretaro University, Mexico. The fungal strains were grown in potato dextrose agar (PDA) medium (Difco Laboratories, Detroit, MI, USA) and incubated at 28 °C for 7 days prior to use. 

The inhibitory effects of VOCs produced by *K. cowanii* Ch1 were evaluated by the inhibition of radial mycelial growth of the fungi using methods described previously [27]. A chamber with two-compartment plastic plates of 9 cm in diameter was used. In the bottom side of the compartment, 100 µL of bacterial suspension (1 × 10^8^ CFU/mL) was inoculated on TSA medium (Difco Laboratories, Detroit, MI, USA). In the top side of th compartment, the PDA medium was added (Difco Laboratories, Detroit, MI, USA) and inoculated with a 7-mm diameter disk of each fungus tested. The negative control only included the fungi tested without bacterial suspension. The chamber with both Petri dishes was immediately sealed with parafilm and incubated for 5–7 days at 28 °C. Then, radial mycelial growth was registered. Each experiment with each fungi strain was performed in triplicate. The following equation was used to analyze the percentage of mycelial growth inhibition (mgi): mgi (%) = [(dc − dt)/dc] × 100, where the terms dc and dt (in mm) represent the average colony diameters of the control and treatment groups, respectively. 

The data obtained were analyzed using the DPS V12.01 software for the analysis of variance and Duncan’s multiple range test (*p* = 0.05) to measure specific differences between pairs of means.

### 2.6. HS–SPME–GC–MS Analysis of VOCs from K. cowanii Ch1

*K. cowanii* Ch1 was inoculated in 8 borosilicate glass media bottles of 80 mL (Schott) with 40 mL of tryptic soy medium, sealed with polypropylene screw caps, and incubated at 37 °C for 48 h with continuous shaking (100 rpm). Tryptic soy medium without the strain was used as a blank control. From each culture, samples were obtained every 6 h to collect cell-free filtrate (FCF) as follows: each sample was centrifugated at 14,000 rpm for 5 min two times and filter sterilized using a membrane with a pore size of 0.2 μm (Sigma-Aldrich, Toluca, Mexico). Mycelial growth inhibition was tested using 500 µL of FCF on PDA medium inoculated with a 7-mm diameter disk of *A. alternata*. An eighteen-hour growth bacterial culture was used for VOC analysis. Samples were incubated at 50 °C for 1 h, and VOCs were collected on a divinylbenzene/carboxen/polydimethylsiloxane fiber (DVB/CAR/PDMS, Supelco, Sigma-Aldrich, Visalia, CA, USA). Manual injection was conducted in splitless mode, and the injection port and transfer line temperature was set to 250 °C using a 7820A GC with a 5975C MSD (Agilent Technologies, Inc., Santa Clara, CA, USA) and HP-5MS 30 m, 0.25 mm, and 0.25 µm GC Column Capillaries (Agilent Technologies Inc., Santa Clara, CA, USA). The column oven was programmed to 40 °C, increasing to 180 °C at 5 °C/min, then 20 °C/min to 260 °C, and held at that temperature for 5 min. Helium (99.999% purity) was used as carrier gas with a flow rate of 1.0 mL/min. Mass spectrometry analyses were conducted using an electron energy of 70 eV, and the *m*/*z* range was 33–500. Data were acquired and processed using NIST/EPA/NIH Mass Spectra Library instrumental analysis software, version 2017, Antioch, CA, USA.

## 3. Results

### 3.1. General Genome Features of Strain K. cowanii Ch1

**Figure 1:**
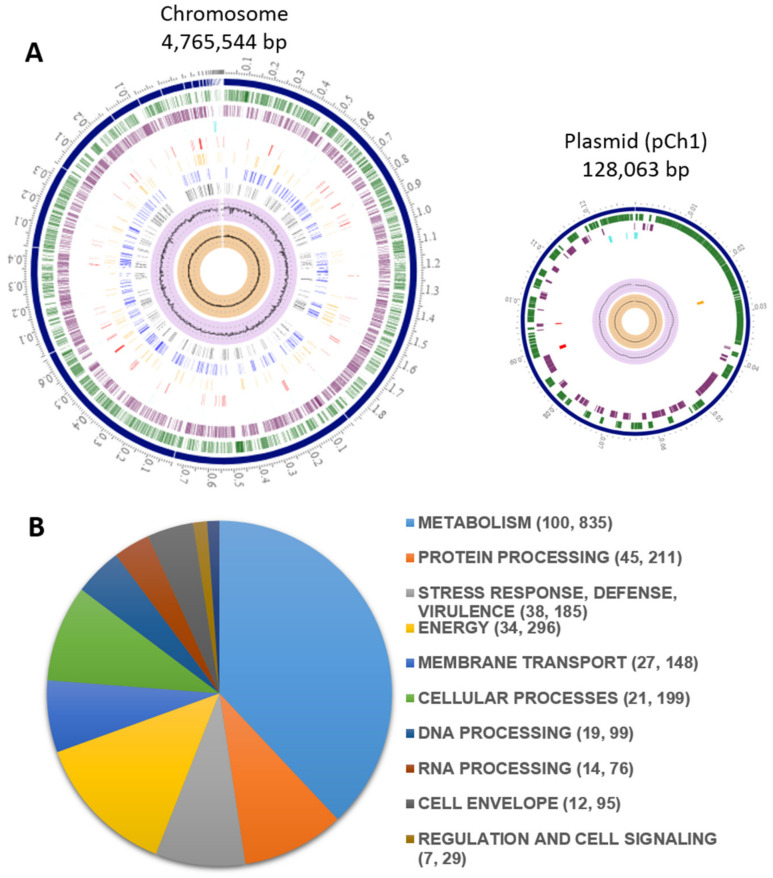
Circular map of *K. cowanii* Ch1 chromosome and plasmid. (**A**) includes, from outer to inner rings, ensembled contigs, CDS on the forward and reverse strand, RNA genes, CDS for antimicrobial resistance genes, CDS for virulence factors, and GC content and skew. (**B**) indicates the subsystem functional assignments. The numbers provided in parentheses are the count of subsystems and genes associated with the subsystem name.

The assembled genome of *K. cowanii* Ch1 had the following statistics: 75 contigs with a total length of 4,765,544 bp, an average G + C content of 56.22%, Contig L50 is nine, Contig N50 is 176,943 bp and a plasmid (pCh1) of 128,063 bp and with an average G + C content of 52.50% (Figure 1A). The genome quality; coarse consistency (98.7), fine consistency (98), CheckM completeness (99.7) and CheckM Contamination (0.1) demonstrate that this genome appears to be of good quality. On this genome we detected 4493 protein coding sequences (CDS) and 90 RNA genes (82 tRNA and 8 rRNA). The annotation included 805 hypothetical proteins and 3688 proteins with subsystem functional assignments (Figure 1B). PATRIC annotation included two types of protein families: 4229 proteins belonging to the genus-specific protein families (PLFams), and 4284 proteins belonging to the cross-genus protein families (PGFams). Many of the genes annotated displayed similarities to known transporters, virulence factors, drug targets, and antibiotic resistance genes. The number of genes and the specific source databases where similarities were found are provided in Table 1. On the plasmid (pCh1) we detected 162 protein coding sequences (CDS), the annotation included 103 hypothetical proteins and 59 proteins with subsystem functional assignments. BLASTN analysis of plasmid pCh1 showed 49% and 46% alignment with plasmid 888-76-1 and plasmid Wem22, respectively; the first plasmid was detected in the pathogenic *K. cowanii* JCM 10956 strain and the second plasmid was detected in *K. cowanii* SMBL-WEM22 strain isolated from seawater in Hong Kong. pCh1 is an IncF plasmid containing *repFIB* gene that encode to a replication protein and the VapBC toxin-antitoxin systems.

**Figure 2:**
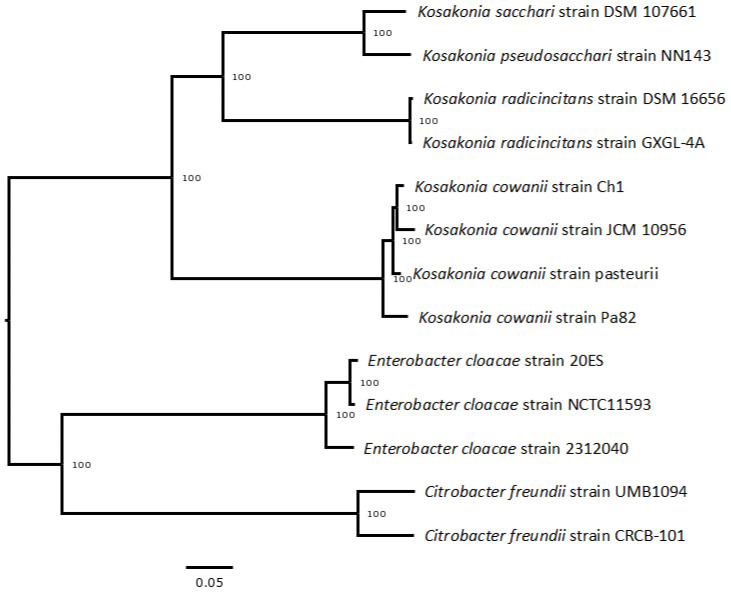
Phylogenetic tree analysis of *K. cowanii* Ch1. The Codon Tree pipeline of BV-BRC was used to generate bacterial phylogenetic trees with *Kosakonia* genus strains (see methodology). *Enterobacter cloacae* and *Citrobacter freundi* were selected as outgroups.

A summary of the antimicrobial resistance genes (AMR) annotated in this genome and the corresponding AMR mechanism classified into eight categories is provided in Table 2. Regarding the AMR phenotype, *K. cowanii* Ch1 showed resistance to the β-lactam antibiotics tested, such as penicillin G, ampicillin, and carbenicillin, likely due to the presence of TEM β-lactamase detected in the chromosome. In addition, resistance was observed for erythromycin, clindamycin, and vancomycin, likely due to the presence of genes classified as efflux pumps conferring antibiotic resistance (Table 2). The *K. cowanii* isolates showed marked susceptibility to the remaining antibiotics tested. Using the complete genome sequences from *K. cowanii* Ch1 and type strains of the *Kosakonia* genus, a phylogenomic tree based on 100 core genes was constructed (Figure 2). The results showed that *K. cowanii* Ch1 is closely related to *K. cowanii* JCM 10956, an isolate from human infection, and clustered closely together with *K. cowanii* strain *pasteuri*, an isolate from insect gut, and Pa82 strain, a phytopathogenic isolate.

### 3.2. Pathogenicity Test

**Figure 3:**
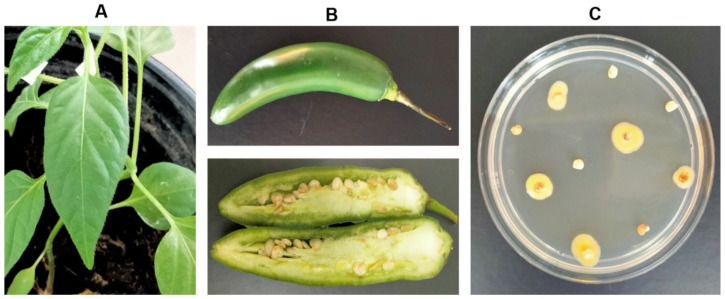
Pathogenesis test. Infection test with *K. cowanii* CH1 is shown in leaves (Panel **A**) and external/internal fruits (Panel **B**) of serrano pepper (*Capsicum annuum* L.). These results were similar to the controls. No lesions were detected when *K. cowanii* was inoculated in plantlets or serrano pepper fruits. Surprisingly, we observed growth of *K. cowanii* Ch1 on seeds of fruits (Panel **C**). TSA medium was supplemented with 100 µg/mL of ampicillin.

The results of the pathogenicity test showed that after inoculation with *K. cowanii* Ch1 in leaves and in external/internal fruits of the serrano pepper (*Capsicum annuum* L.), there were no symptoms of infection (Figure 3A,B). Virulence-related genes identified in the chromosome were principally flagellar, iron uptake, and siderophore components (Table 3), which likely are related to bacterial colonization, but not to pathogenesis in leaves and fruits of the infected plants, since no SSIII or vir/avr genes were identified. As *K. cowanii* may have the capability of invasion and motility due to the presence of genes related to virulence factors identified in the chromosome (*fliP*, *fliM*, *fliG*, *fliC*, *fliA*, *flgH*, *flgG*, *flgC*, *flgB*), we aimed to determine if the bacterium had the capability of colonization inside the fruit tissues. Interestingly, when we analyzed seeds from fruits inoculated with *K. cowanii* Ch1 seven days after inoculation (supplemented with ampicillin to avoid bacterial contamination), approximately 50% of the seeds tested showed growth of *K. cowanii* Ch1 (Figure 3C). This was validated by PCR for 16S rDNA and amplicon sequencing. This result showed that *K. cowanii* Ch1 may spread throughout the seeds of *Capsicum annuum* L.; however, additional research is necessary to understand the chemotactic mechanisms.

### 3.3. Inhibitory Effects of VOCs Produced by K. cowanii Ch1 on Pathogenic Fungi In Vitro

**Figure 4:**
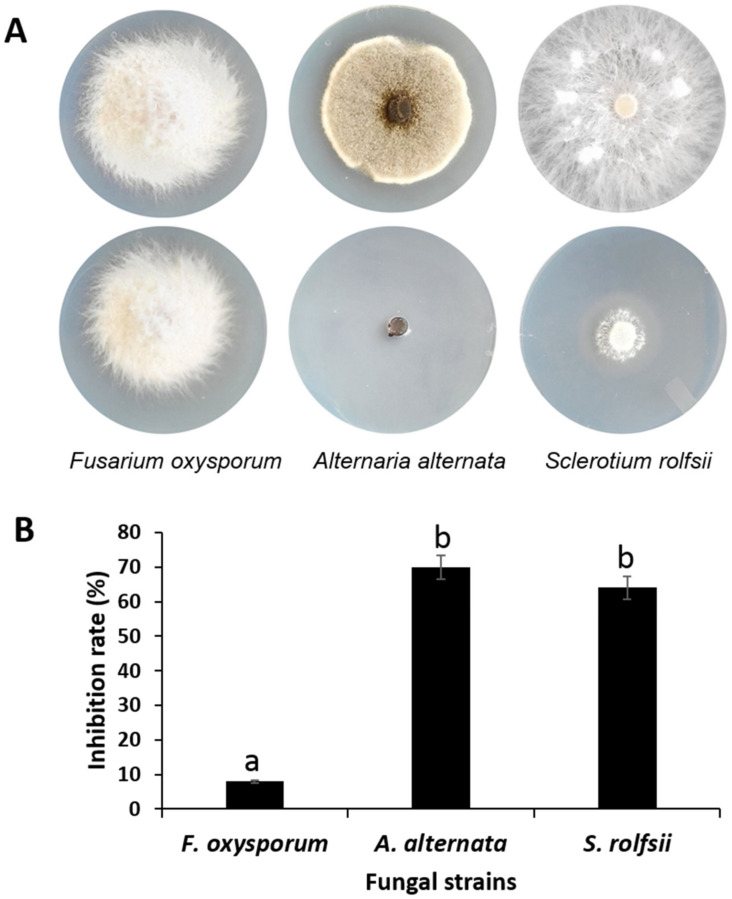
Antifungal activity test of *K. cowanii* Ch1 against fungal pathogens. (**A**) shows radial mycelial growth for controls (upper) and treatment (lower) at the end of experiments. (**B**) shows inhibition rate (%) for fungal strains tested. Values by different letters show statistical differences according to Duncan’s multiple range test (*p* = 0.05).

Given the absence of vir/avr genes in *K. cowanii* Ch1 and the fact that it is not able to produce symptoms of infection in serrano peppers, we wondered if there was any biological activity for this bacterial strain. To answer this question, we initially chose to evaluate potential biocontrol for volatile organic compounds (VOCs) produced during growth condition in the TSA medium and their potential effects on the presence of fungal strains as indicated in the methodology. Surprisingly, the results demonstrated that *K. cowanii* Ch1 presented antifungal abilities against *Sclerotium rolfsii* and *Alternaria alternata* with a mean rate of 64% and 70%, respectively, of inhibition of radial mycelial growth during the period evaluated, while radial mycelial growth inhibition on *Fusarium oxysporum* was lower than 10% (Figure 4). Therefore, we determined that the *K. cowanii* CH1 strain produces some volatile organic compounds with antifungal activity that cause mycelial growth inhibition on *A. alternate* and *S. rolfsii*, and the next step was the identification of these key VOCs produced by this bacterial strain.

### 3.4. HS–SPME–GC–MS Analysis of VOCs from K. cowanii Ch1

**Figure 5:**
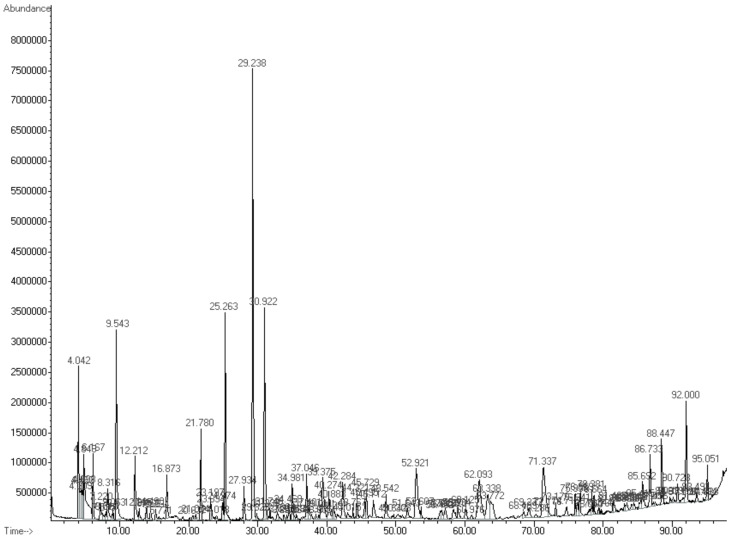
VOC detection from 18 h of fermentation culture of *K. cowanii* Ch1 using HS–SPME–GC–MS. Abundance and retention time are shown for each peak.

To explore the fungal inhibition rate of VOCs produced by *K. cowanii* Ch1 during different fermentation times, filtrate cell free (FCF) values were obtained every 6 h for a 48 h period. The results showed that the FCF values at 18 h exhibited the highest mean inhibition rate against *A. alternata*, with a mean rate of 60%, while the FCF values at 12 h had only a mean rate of fungal inhibition of 20%; the FCF results from other fermentation times showed no significant inhibition. Therefore, we decided to analyze the VOCs from the fermentation time at 18 h using HS–SPME–GC–MS (Figure 5).

The volatile organic compounds detected in the control were compared with those produced by *K. cowanii* Ch1 according to its relative peak area, and the results showed 67 VOCs (Table 4). The chemical classes detected were acids (3), alcohols (14), aldehydes (5), esters (11), hydrocarbons (6), ketones (11), organochlorines (2), aromatics (3), thioethers (3), and pyrazines (9). As is indicated by the relative peak area of the HS–SPME–GC–MS profile, the nine compounds with the highest area percentages for each chemical class were dodecanoic acid, 3-hydroxy (1.08%); ethanol (5.40%); 1-butanol-3-methyl (4.88%); acetaldehyde (3.81%); butanoic acid, butyl ester (3.86%); cyclodecane (8.14%); 2-butanone, 3-hydroxy (10.60%); disulfide, dimethyl (1.01%); and pyrazine-2,5-dimethyl (5.86%). Although individual or mixes of synthetic compounds were not tested among the pathogenic fungi, some of them were similar to the VOCs detected in other bacteria with inhibitory activity against pathogenic fungi [27].

## 4. Discussion

The *Kosakonia* genus possess a versatile metabolic ability to colonize different ecological niches with diverse beneficial or detrimental effects [2,3,4,5,6,7,8,9,10,13,28]. Thus, environmental persistence through all of these abiotic stress tolerance features, such as the ability to grow in a wide range of temperatures, pH values, and salt concentrations [11], makes it possible to explain why *Kosakonia* was detected in chili powder [29]. The research approach developed in this study enabled the isolation and characterization of a new strain of nonpathogenic *K. cowanii* Ch1, at least for serrano pepper (*Capsicum annuum* L.), with an important metabolic ability to inhibit mycelial growth in pathogenic fungi through the production during fermentation of volatile organic compounds (VOCs). 

According to the genome sequencing and phylogenetic analysis, *K. cowanii* Ch1 appeared to be the closest to *K. cowanii* JCM 10956, a human pathogen isolated from human blood [2], and in the same clade as the phytopathogenic *K. cowanii* Pa82 [5]. *K. cowanii* JCM 10956 has two plasmid and virulence-related genes with a type III secretion system [2]. Interestingly, plasmid pCh1 has a 49% of similarity with plasmid p888-76-1 of *K. cowanii* JCM 10956 strain [2] and 46% of similarity with plasmid Wem22 identified in *K. cowanii* SMBL-WEM22 strain isolated from seawater in Hong Kong [30]. On the other hand, *K. cowanii* Pa82 has three plasmid and virulence-related genes with a type VI secretion system. In fact, in the *K. cowanii* Pa82 strain, the mutation of the *vgrG* gene, which encodes one of the type VI secretion system components, was functionally validated as a virulence factor causing patchouli bacterial wilt [5]. In Gram-negative bacteria, the type VI secretion system participates in diverse functions, including virulence, antibacterial activity, and metal-ion uptake, conferring advantages during bacterial colonization [31]. On the other hand, genome sequencing revealed that *K. cowanii* Ch1 has virulence-related genes of invasion, such as flagellar component genes, but not for secretion systems nor for *vir*/*avr* genes (Table 3), which might explain the absence of infection symptoms in the leaves and fruits of the serrano pepper (*Capsicum annuum* L.) when the pathogenicity test was carried out. However, the colonization of serrano pepper seeds by *K. cowanii* Ch1 may lead to an important route for the spread of the bacterium and is likely related to the presence of *K. cowanii* in chili powder [29]; future investigation may reveal these chemotaxis mechanisms. Additionally, complete genome sequencing of *K. cowanii* Ch1 revealed the presence of antimicrobial resistance genes (Table 2), which are likely related to the resistance mechanisms of the β-lactam, erythromycin, clindamycin, and vancomycin antibiotics tested; thus, the potential public health risks for this bacterial strain must be evaluated due to its presence in chili powder. Therefore, the genomic information of *K. cowanii* Ch1 will be important for clarifying and understanding the evolution of metabolic versatility of the *Kosakonia* genus.

In recent decades, the increasing use of bacterial inoculants for plant growth promotion and biocontrol of phytopathogenic microorganisms has allowed for the isolation and characterization of the *Kosakonia* genus. Thus, for example, *K. pseudosacchari* TL8 and *K. pseudosacchari* TL13 have been characterized with antimicrobial activity against phytopathogens, such as *Botrytis* spp. and *Phytophthora* spp., as well as with multiple plant growth promotion activities and abiotic tolerance traits [11]. In a different study, *K. cowanii* B-6-1 [12] was isolated from tomato, and characterization showed biocontrol activity against *F. verticillioides*, *A. tenuissima*, and *B. cinerea*. However, in these studies, the production and identification of VOCs as biocontrols of these fungi were not conducted. 

Recent studies have demonstrated the potential of VOCs during plant and post-harvest disease control caused by pathogenic microorganisms [28,32]. Diverse compounds of different chemical classes of VOCs, such as alkenes, alcohols, ketones, organic acids, terpenes, benzenoids, and pyrazines, have been characterized, and based on their physical–chemical properties it has been established that they may affect cell walls and membranes through the downregulation of gene expression related to cell membrane fluidity, wall integrity, energy metabolism, and the production of cell wall-degrading enzymes, as well as alteration in the redox balance [32]. Therefore, in this study, the discovery of the *K. cowaniii* Ch1 strain with capability of biocontrol in vitro of *A. alternata* and *S. rolfsii*, likely due to VOC production, provides an additional mechanism for *Kosakonia* genus biocontrol activity. Of the 67 potential VOCs identified, nine compounds (dodecanoic acid, 3-hydroxy; ethanol; 1-butanol-3-methyl; acetaldehyde; butanoic acid, butyl ester; cyclodecane; 2-butanone, 3-hydroxy; disulfide, dimethyl; and pyrazine-2,5-dimethyl) were the most abundant compounds. Interestingly, these compounds have been detected in other bacteria with antifungal or antibacterial effects [28,32]; however, cyclodecane is not listed in the mVOC 2.0 Database [33]. Nevertheless, the variation, concentration, and antifungal effects of VOCs produced by each bacterial strain reported are specific, probably due to microbial growth conditions, nutritional requirements, genetics features, and mechanisms, with multiple modes of action against specific pathogenic microorganisms. Therefore, the mycelial growth inhibitory effects on *A. alternata* and *S. rolfsii*, but not *F. oxysporum*, cannot be easily explained through the effects of a single compound from a heterogeneous mix of VOCs.

The production of 2-butanone, 3-hydroxy (acetoin), for example, showed the highest relative peak area in *K. cowaniii* Ch1. According with genome analysis in *K. cowanii* Ch1, the presence of acetolactate decarboxylase could be an important metabolic route to obtain acetoin and further transformed by reversible reduction into 2, 3-butanediol by 2,3-butanediol dehydrogenase, also present in the genome. Also, production of 2,3-butanedione detected in *K. cowanii* Ch1 (Table 4) from nonenzymatic oxidative decarboxylation of acetolactate and converted to acetoin and 2,3-butanediol by 2,3-butanediol dehydrogenase could be important. Acetoin compound has been detected in some bacteria, such as the *Bacillus* and *Paenibacillus* genera, affecting mycelial growth and spore germination inhibition against diverse fungal strains [28]. Furthermore, the Enterobacteriaceae family commonly produces acetoin from pyruvate during the fermentation of glucose or other carbon sources through the Embden–Meyerhof (EM) pathway [34] and has garnered attention because they function as signal molecules in plants, promoting growth, drought tolerance, and defense against pathogens [35,36,37]. Additionally, it has been suggested that the acetoin pathway is related to plant colonization of pathogenic microorganisms and as pheromones to attract a wide variety of insects [34]. On the one hand, the alcohol class detected in *K. cowanii* Ch1, such as ethanol and 1-butanol-3-methyl, with high relative peak areas, are also commonly produced during fermentation in the Enterobacteriaceae family and have been previously detected as components of VOCs in bacterial and fungal strains with antifungal activity [28]. The presence of aldehyde dehydrogenase and alcohol dehydrogenase enzymes in the genome of *K. cowanii* Ch1 represent an important key to convert the precursor acetyl-CoA to ethanol via acetaldehyde route during ethanol fermentation in an anaerobic environment. Also, butanal (butyraldehyde) route was detected in *K. cowanii* Ch1 genome and might lead to obtaining butanol [34]. Pyrazine-2,5-dimethyl is another VOC found in *K. cowanii* Ch1 with a high relative peak area and has been reported in diverse bacterial species. According to the genome analysis, this is synthetized probably through a threonine pathway to convert the precursor amino acetoacetate. Also, this compound can significantly inhibit the growth of plant pathogens such as *Magnaporthe oryzae*, *Phytophthora capsici*, and *A. solani* [32]. On the other hand, it has been reported that *Pseudomonas*, *Serratia*, *Bacillus*, and *Stenotrophomonas* produce disulfide, dimethyl as the most abundant compound in their VOC mixtures, with the ability to control the growth of a wide range of plant pathogens [38], and in our case was produced and detected in *K. cowanii* Ch1. In addition, disulfide, dimethyl improves the growth of plants and induces systemic resistance against plant pathogens [38]. In general, additional VOCs were produced, although with lower abundance, such as acids, alcohols, aldehydes, esters, hydrocarbons, ketones, organochlorines, aromatics, thioethers, and pyrazines, which have been reported to have antifungal activity according to the mVOC 2.0 Database [33].

In vitro experiments confirmed that *K. cowanii* Ch1 has antifungal activity against *A. alternata* and *S. rolfsii*, but not against *F. oxysporum*, probably due to VOCs produced with multiple routes of cell affectation, which accords with the diverse literature demonstrating the potential of VOCs against diverse pathogenic microorganisms. However, we did not perform single compound evaluations, since several reports had evaluated in vitro and in vivo single VOCs with different results, including use limitations on field crops and toxic effects in plants and animals [28,32]. Therefore, additional experiments are required to demonstrate the potential ecological role of *K. cowanii* Ch1 on plant responses against phytopathogenic microorganisms that accord with some of the VOCs detected in our analysis.

In conclusion, the genome sequencing of *K cowanii* Ch1 open the possibility to further exploring important metabolic pathways related to VOC production and microbial responses. Additionally, the finding that *K. cowanii* Ch1 may spread through chili seeds but cause no infection in fruits and leaves of *Capsicum annuum* L., likely due to the absence of virulence-related genes of the secretion system, opens up the possibility that this bacterial strain can be used as an excellent candidate for the development of biocontrol agents. However, further studies are still needed to understand the role of the VOCs produced by *K. cowanii* Ch1 on pathogenic microorganisms and plant responses, as well as an antibiotic resistance analysis for biosafety during potential applications as a biocontrol.

## Figures and Tables

**Table 1:** Specialty genes.

Category	Source	Genes
Antibiotic Resistance	CARD	49
Antibiotic Resistance	PATRIC	60
Drug Target	DrugBank	275
Drug Target	TTD	54
Drug Target	TCDB	458
Virulence Factor	PATRIC_ VF	103
Virulence Factor	VFDB	25
Virulence Factor	Victors	128

**Table 2:** Antimicrobial resistance genes.

AMR Mechanism	Genes
Antibiotic activation enzyme	*katG*
Antibiotic resistance gene cluster, cassette, or operon	*marA*, *marB*, *marR*, *bla*
Antibiotic target in susceptible species	*alr*, *ddl*, *drx*, *EF-G EF-Tu*, *folA*, *dfr*, *folP*, *gyrA*, *gyrB*, *inhA*, *fabl*, *lso-Trna*, *kasA*, *murA*, *rho*, *rpoB*, *rpoC*, *s10p*, *S12p*
Antibiotic target protection protein	*BcrC*
Efflux pump conferring antibiotic resistance	*acrAB-tolC*, *acrAD-tolC*, *acrZ*, *emrAB-tolC*, *macA*, *macB*, *mdfA/cmr*, *mdtABC-tolC*, *sugE*, *tolC/opmH*
Gene conferring resistance via absence	*GidB*
Protein-altering cell-wall charge conferring antibiotic resistance	*gdpD*, *pgsA*
Regulator modulating expression of antibiotic resistance genes	*acrAB-tolC*, *emrAB-tolC*, *H-NS*, *oxyR*

**Table 3:** Potential virulence-related genes in *K. cowanii* Ch1 predicted by VFDB.

Virulence Factor	Gene Name	Putative Function
Endotoxin	*gtrB*	Bactoprenol glucosyl transferase
Iron uptake, siderophore	*entA*	2,3-dihydro-2,3-dihydroxybenzoate dehydrogenase 2,3-dihydro-2,3-dihydroxybenzoate dehydrogenase of siderophore biosynthesis
	*fepB*	Ferric enterobactin-binding periplasmic protein FepB
	*entS*	Enterobactin exporter EntS
	*fepG*	Ferric enterobactin transport system permease protein FepG
	*entB*	Isochorismatase of siderophore biosynthesis
Secretion system, invasion, motility	*flgC*	Flagellar basal-body rod protein FlgC
	*motA*	Flagellar motor rotation protein MotA
	*flgH*	Flagellar L-ring protein FlgH
	*flgB*	Flagellar basal-body rod protein FlgB
	*flgG*	Flagellar basal-body rod protein FlgG
	*fliG*	Flagellar motor switch protein FliG
	*fepD*	Ferric enterobactin transport system permease protein FepD
	*fliP*	Flagellar biosynthesis protein FliP
	*cheW*	Positive regulator of CheA protein activity (CheW)
	*fliM*	Flagellar motor switch protein FliM
	*fliC*	Flagellin FliC
	*fliA*	RNA polymerase sigma factor for flagellar operon

**Table 4:** VOCs produced by *K. cowanii* Ch1 detected by HS–SPME–GC–MS.

Compounds	Retention Time (min)	Relative Peak Area (%)	Chemical Classes	Compounds	Retention Time (min)	Relative Peak Area (%)	Chemical Classes
Ethyl ether	4.463	1.21	Esters	1-Hexanol, 2-ethyl-	39.375	2.53	Alcohols
Methanethiol	4.698	0.72	Alcohols	Benzaldehyde	40.274	1.04	Aldehydes
Acetaldehyde	4.849	3.81	Aldehydes	1-Octanol	42.284	1.74	Alcohols
Acetone	6.167	0.87	Ketones	2-Undecanone	44.321	0.70	Ketones
Butanal	7.595	0.26	Aldehydes	2-Acetylthiazole	45.495	0.53	Other compounds
Ethyl Acetate	8.016	0.23	Esters	Acetophenone	45.729	0.68	Ketones
2-Butanone	8.316	0.84	Ketones	Pyrazine, 2,5-dimethyl-3-(3-methylbutyl)-	46.712	0.80	Pyrazine
Butanal, 3-methyl-	9.063	0.23	Aldehydes	Acetic acid, decyl ester	48.542	0.97	Esters
Ethanol	9.543	5.40	Alcohols	2-Tridecanol	50.308	0.25	Alcohols
2,3-Butanedione	12.212	2.15	Ketones	Cyclodecane	52.921	8.14	Hydrocarbons
Trichloroethylene	12.835	0.39	Other compounds	2-Tridecanone	57.159	0.56	Ketones
Trichloromethane	14.49	0.40	Other compounds	3-Decen-1-ol, acetate, (Z)-	58.32	0.65	Alcohols
Toluene	15.234	0.36	Hydrocarbons	Benzyl Alcohol	60.125	0.54	Alcohols
Disulfide, dimethyl	16.873	1.01	Other compounds	Butanoic acid, butyl ester	62.093	3.86	Esters
p-Xylene	21.054	0.16	Other compounds	Propanoic acid, 2-methyl-, 2,2-dimethyl-1-(2-hydroxy-1-methylethyl)propyl ester	63.338	1.89	Esters
1-Butanol	21.78	1.74	Alcohols	Phenylethyl Alcohol	63.772	1.66	Alcohols
Ethanol, 2-methoxy-	23.334	0.32	Alcohols	Ethanol, 2,2′-oxybis-	68.499	0.36	Alcohols
2-Heptanone	24.018	0.10	Ketones	2-Propenal, 3-phenyl-	73.17	0.35	Aldehydes
1,3-Diazine	24.974	0.50	Other compounds	2-Nonadecanone	74.715	0.50	Ketones
1-Butanol, 3-methyl-	25.263	4.88	Alcohols	Octanoic Acid	76.241	0.59	Acids
Pyrazine, methyl-	27.934	1.06	Pyrazine	Triacetin	76.669	0.60	Other compounds
2-Butanone, 3-hydroxy-	29.238	10.60	Ketones	Cyclododecane	78.05	0.36	Hydrocarbons
2-Propanone, 1-hydroxy-	29.823	0.23	Ketones	2,4,7,9-Tetramethyl-5-decyn-4,7-diol	78.381	1.10	Alcohols
Pyrazine, 2,5-dimethyl-	30.922	5.86	Pyrazine	Benzoic acid, 4-tert-butyl-, 3,5-dichloro-4-pyridyl ester	79.344	0.23	Esters
Pyrazine, 2,6-dimethyl-	31.233	0.25	Pyrazine	Benzoic acid, 2-amino-, methyl ester	83.028	0.27	Esters
1-Hexanol	32.78	0.33	Alcohols	Cyclododecene	84.094	0.30	Hydrocarbons
Dimethyl trisulfide	33.755	0.16	Other compounds	Methyl 8-methyl-nonanoate	84.442	0.24	Esters
Pyrazine, 2-ethyl-6-methyl-	34.201	0.11	Pyrazine	Hexanedioic acid, dibutyl ester	85.415	0.35	Esters
Pyrazine, 2-ethyl-5-methyl-	34.469	0.40	Pyrazine	Dodecanoic acid, 3-hydroxy-	85.692	1.08	Acids
2-Nonanone	34.981	1.00	Ketones	Diethyl Phthalate	88.447	1.39	Other compounds
Pyrazine, 2-methyl-5-(1-methylethyl)-	35.581	0.13	Pyrazine	Indole	90.111	0.15	Aromatics
Pyrazine, 3-ethyl-2,5-dimethyl-	37.046	1.28	Pyrazine	Homosalate	92	3.09	Other compounds
Octanoic acid, ethyl ester	37.401	0.43	Esters	Phthalic acid, hex-2-yn-4-yl isobutyl ester	95.051	0.89	Other compounds
Pyrazine, 2-ethenyl-6-methyl-	38.835	0.09	Pyrazine				

## Data Availability

The data presented in this work are available from the corresponding authors upon request.
